# α-TEA cooperates with chemotherapeutic agents to induce apoptosis of p53 mutant, triple-negative human breast cancer cells via activating p73

**DOI:** 10.1186/bcr2801

**Published:** 2011-01-07

**Authors:** Richa Tiwary, Weiping Yu, Bob G Sanders, Kimberly Kline

**Affiliations:** 1 School of Biological Sciences/C0900, University of Texas, 1 University Station, Austin, TX 78712, USA; 2 Department of Nutritional Sciences/A2703, University of Texas, University Station, Austin, TX 78712, USA

## Abstract

**Introduction:**

Successful treatment of p53 mutant, triple-negative breast cancers (TNBC) remains a daunting challenge. Doxorubicin (DOXO) and cisplatin (CDDP) are standard-of-care treatments for TNBC, but eventually fail due to acquired drug resistance and toxicity. New treatments for overcoming drug resistance and toxicity in p53 mutant, TNBC are therefore badly needed. Unlike p53, p73 - a member of the p53 family - is usually not mutated in cancers and has been shown to regulate p53-mediated apoptotic signaling in p53-deficient cancers. Therefore, identification of anticancer agents that can activate p73 in p53-deficient cancers may provide a chemotherapeutic approach for treatment of p53 mutant cancers. Here we report on the reconstitution of the p53 tumor suppressor pathway in a p53-independent manner via p73 with combination treatments of α-TEA, a small bioactive lipid, plus DOXO or CDDP.

**Methods:**

p53 mutant, TNBC cell lines MDA-MB-231, BT-20 and MDA-MB-468 were used to evaluate the anticancer effect of chemotherapeutic drugs and α-TEA using annexin V (FITC)/PI staining, western blot analyses, RT-PCR and siRNA knockdown techniques.

**Results:**

Combination treatments of α-TEA plus DOXO or CDDP act cooperatively to induce apoptosis, caspase-8 and caspase-9 cleavage, p73, phospho-c-Ab1 and phospho-JNK protein expression, and increase expression of p53 downstream mediators; namely, death receptor-5, CD95/APO-1 (Fas), Bax and Noxa, as well as Yap nuclear translocation - plus reduce expression of Bcl-2. Knockdown of p73, c-Abl, JNK or Yap using siRNAs shows that p73 plays a critical role in combination treatment-enhanced apoptosis and the expression of pro-apoptotic and anti-apoptotic mediators, and that c-Abl, JNK and Yap are upstream mediators of p73 in combination treatment responses.

**Conclusions:**

Data show that α-TEA in combination with DOXO or CDDP synergistically enhances apoptosis in TNBC via targeting p53-mediated genes in a p73-dependent manner, and that p73 responses are downstream of c-Abl, JNK and Yap.

## Introduction

Successful treatment of triple-negative breast cancers (TNBC) (estrogen receptor (ER)-negative, progesterone receptor-negative and Her-2-negative), that are also p53 mutant remains elusive. Unfortunately, the anticancer efficacy of commonly used chemotherapeutic agents for TNBC, including doxorubicin (DOXO) and cisplatin (CDDP), are limited due to acquired drug resistance and toxicities [1,2].

DOXO and CDDP are DNA-damaging drugs that exert their anticancer actions via inhibition of cellular proliferation and induction of cell death by apoptosis [3,4]. The tumor suppressor gene p53 plays a central role in the anticancer actions of DNA-damaging agents. Loss of wild-type p53 functions leads to resistance to DNA-damaging agents, such as DOXO and CDDP [5,6]. Identification of anticancer agents that target p53 downstream genes via p53-independent mechanisms is of major clinical relevance, especially since p53 deficiency is a hallmark of many different cancer types.

p73 is a member of the p53 gene family [7]. Unlike p53 [8], p73 is rarely mutated or lost in cancers [9]. Although p53-deficient cancers are less responsive to chemotherapy, they are typically not completely drug resistant because other p53 family members, such as p73, can replace p53 function in response to DNA damage [9-11]. Since p73 is usually not mutated in cancers and has been shown to regulate p53 target genes in p53-deficient cancers, identification of anticancer agents that can activate p73 in p53-deficient cancers will provide a chemotherapeutic approach for treatment of drug-resistant p53 mutant cancers.

α-TEA (2,5,7,8-tetramethyl-2R-(4R,8R,12-trimethyltridecyl) chroman-6-yloxyacetic acid, known as RRR-α-tocopherol ether-linked acetic acid analog or RRR-α-tocopheryloxyacetic acid) is a nonhydrolyzable ether analog of RRR-α-tocopherol [12]. α-TEA has been shown to be a potent pro-apoptotic agent both in *vitro *and *in vivo *in breast, prostate and ovarian cancer cells [12-20]. Recently, α-TEA has been shown to delay tumor onset and to inhibit the progression and metastatic spread in a clinically relevant model of spontaneous mammary cancer, further highlighting the translational potential of this anticancer agent [14]. Mechanisms involved in α-TEA-induced apoptosis include activation of JNK/c-Jun, p73/NOXA and Fas/death receptor-5 (DR5), and suppression of c-FLIP-L, survivin and phospho-Akt (pAkt) - leading to death receptor-mediated caspase-8 activation and mitochondria-dependent apoptosis [15-20].

Data presented here show that α-TEA in combination with DOXO or CDDP significantly enhances apoptosis of p53 mutant, triple-negative human breast cancer cells by targeting p73-mediated p53-dependent pro-apoptotic and anti-apoptotic genes via c-Abl, JNK and Yap signaling pathways.

## Materials and methods

### Chemicals

α-TEA was made in-house as previously described [12]. DOXO and CDDP were purchased from Sigma (San Diego, CA, USA). Phosphoinositide 3-kinase inhibitor (wortmannin) was purchased from Cell Signaling Technology (Beverly, MA, USA).

### Cell culture

p53 mutant, triple-negative human breast cancer cell lines MDA-MB-231, BT-20 and MDA-MB-468 were purchased from the American Type Culture Collection (Manassas, VA, USA). MDA-MB-231 and BT-20 cells were cultured in MEM media with 10% FBS, and MDA-MB-468 cells were cultured in Dulbecco's MEM media with 10% FBS. All three p53 mutant TNBC cell lines (ER^-^, PR-^-^, HER2^-/low^) used in these studies were originally obtained from human samples so no isogenic counterparts expressing wildtype p53, ER and progesterone receptor are available for use as controls. For experiments, FBS was reduced to 2% to better mimic low *in vivo *serum exposure and cells were allowed to attach overnight before treatment. α-TEA (40 mM) was dissolved in ethanol as a stock solution. Concentrations of ethanol used in vehicle treatments were 0.025 to 0.05% (v/v) to match the ethanol content in the different final concentrations of α-TEA treatments. DOXO and CDDP were dissolved in H_2_O.

### Quantification of apoptosis

Apoptosis was quantified by annexin V-FITC/PI assays following the manufacturer's instructions. Fluorescence was measured using fluorescence-activated cell sorter analyses with a FACSCalibur flow cytometer, and data were analyzed using CellQuest software (BD Biosciences, San Jose, CA, USA). Cells displaying phosphatidylserine on their surface (that is, positive for annexin-V fluorescence) were considered apoptotic.

### Nuclear and cytoplasmic fractionation

Cytoplasmic and nuclear fractions were prepared as previously described [21]. Briefly, whole cell lysates were centrifuged to obtain supernatant and pellet. The supernatant was centrifuged again and the resulting supernatant was used as the cytosolic fraction. The pellet was layered over a cushion of 1 ml sucrose buffer and centrifuged. The final pellet, lysed using RIPA buffer, was used as the nuclear fraction.

### Western blot analyses

Whole cell protein lysates were prepared and western blot analyses were conducted as described previously [22]. Proteins (20 to 50 μg/lane) were separated by SDS-PAGE and transferred to nitrocellulose (Optitran BA-S-supported nitrocellulose; Schleicher and Schuell, Keene, NH, USA). Antibodies to the following proteins were used: poly(ADP-ribose) polymerase (PARP), Fas, Bcl-2, Bax, total JNK and phospho-JNK (pJNK) (Santa Cruz Biotechnology, Santa Cruz, CA, USA); p73 and NOXA (Imgenex, San-Diego, CA, USA); and pYap (Ser-127), Yap, p-cAbl (Tyr-245), c-Abl, pAkt (Ser-473), caspase-8, caspase-9, DR5 and glyceraldehyde-3-phosphate dehydrogenase (Cell Signaling Technology).

### RT-PCR detection of Fas, DR5, Bax, Noxa and Bcl-2 mRNA expression

Total RNA was extracted using an RNA isolation kit (Qiagen Inc., Valencia, CA, USA). Semi-quantitative analyses were conducted to detect Fas, DR5, Bax, Noxa and Bcl-2 mRNA by RT-PCR using the housekeeping gene β-actin as control. Total RNA was reverse transcribed to cDNA using Superscript RTase (250 U; Invitrogen, Carlsbad, CA, USA) following the manufacturer's instructions. cDNA was used per PCR reaction with Taq PCR Master Mix Kit (Qiagen Inc.) plus 10 μM oligonucleotide primer pairs (Invitrogen). The primer sequences are presented in Table 1.

**Table 1:** Primer sequences

Gene	Forward primer	Reverse primer
Fas	5'-CAATGGGGATGAACCAGACTGC-3'	5'-GGCAAAAGAAGAAGACAAAGCC-3'
DR5	5'-GCCTCATGGACAATGAGATAAAGGTGGCT-3'	5'-CCAAATCTCAAAGTACGCACAAACGG-3'
Bax	5'-AGTAACATGGAGCTGCAGAGGATG-3'	5'-AGGAGGCTTGAGGAGTCTCACC-3'
Noxa	5'-CGTGTGTAGTTGGCATCTCC-3'	5'-AAGGAGTCCCCTCATGCAAG-3'
Bcl-2	5'-CCTGTGGATGACTGAGTACC-3'	5'-GAGACAGCCAGGAGAAATCA-3'
β-actin	5'-GGCGGCACCACCATGTACCCT-3''	5'-AGGGGCCGGACTCGTCATACT-3'

### RNA interference

A scrambled RNA duplex purchased from Ambion (Austin, TX, USA) that does not target any known mouse, rat or human gene was used as the nonspecific negative control for interfering RNA (referred to as control siRNA). Transfection of MDA-MB-231 cells with siRNA to p73, c-Abl, JNK, Yap or control (Ambion) was performed in 100-mm cell culture dishes at a density of 2 × 10^6 ^cells/dish using Lipofectamine 2000 (Invitrogen) and siRNA duplex, resulting in a final siRNA concentration of 30 nM following the company's instructions. After 1 day of exposure to transfection mixture, the cells were re-cultured in a 100-mm dish at 2 × 10^6 ^cells/dish and incubated for 1 day followed by treatment.

### Statistical analysis

Apoptosis data were analyzed using one-way analysis of variance followed by the Tukey test for comparison of more than two treatments or a two-tailed Student *t *test for comparison between two treatments to determine statistical differences. Differences were considered statistically significant at *P *< 0.05.

## Results

### α-TEA, DOXO and CDDP induce apoptosis in p53 mutant, human TNBC cells

The sensitivity of three p53 mutant, TNBC lines (MDA-MB-231, BT-20 and MDA-MB-468) to apoptosis induced by α-TEA, DOXO and CDDP was evaluated by determining half-maximal effective concentration values for apoptosis (Table 2). Data show that MDA-MB-468 cells exhibit the most sensitive phenotype and MDA-MB-231 cells exhibit the most resistant phenotype to apoptosis induced by DOXO and CDDP among the three cell lines. The sensitivity of the three cell lines to α-TEA-induced apoptosis, however, is similar.

**Table 2:** Half-maximal effective concentration values for apoptosis

Cell line	α-TEA (μM)	DOXO (μM)	CDDP (μM)
MDA-MB-231	41.7	46.5	70.8
BT-20	45.4	25.7	64.0
MDA-MB-468	35.4	8.5	40.7

Cells were treated with different concentrations of α-TEA, doxorubicin (DOXO), and cisplatin (CDDP) for 24 hours. Apoptosis was determined by annexin V-FITC/PI staining/fluorescence-activated cell sorter analysis as described in Material and methods. The concentration that achieved 50% apoptosis (half-maximal effective concentration) was determined using commercially available software (Calcusyn; Biosoft, Manchester, UK).

### α-TEA cooperates with DOXO and CDDP to induce apoptosis of p53 mutant, TNBC cells

**Figure 1:**
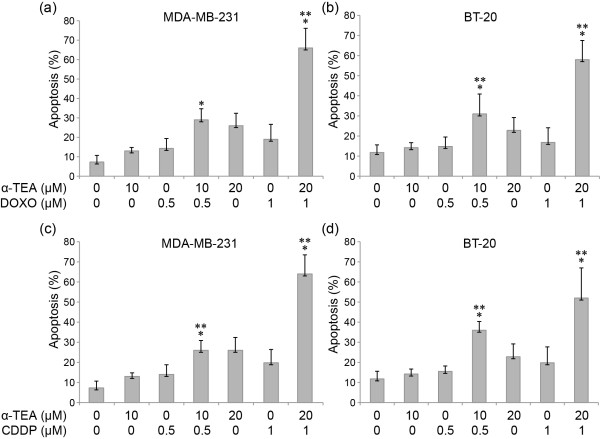
**α-TEA, doxorubicin and cisplatin induce apoptosis of p53 mutant, triple-negative human breast cancer cells**. MDA-MB-231 and BT-20 breast cancer cells were treated with different concentrations of doxorubicin (DOXO) or cisplatin (CDDP) alone or in combination with α-TEA for 24 hours. Fluorescence-activated cell sorter/annexin V assays were used to determine the percentage of apoptotic cells **(a), (b), (c), (d)**. Data expressed as mean ± standard deviation from three independent experiments. *P <0.05, significantly different from control. **P <0.05, significantly different from single treatments.

**Figure 2:**
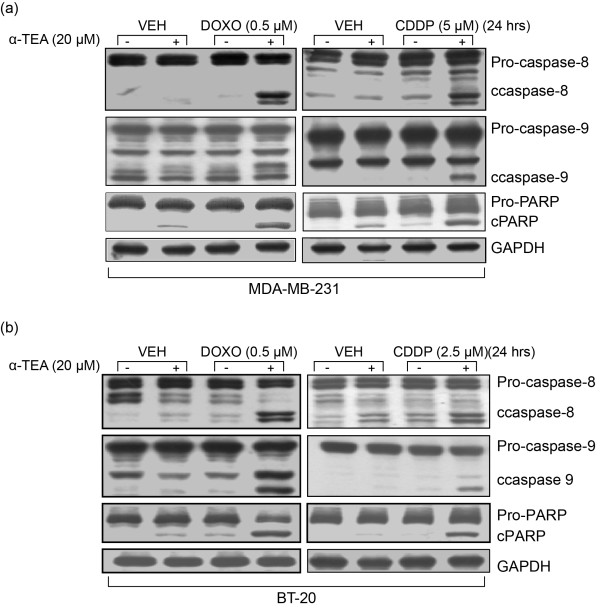
**α-TEA cooperates with doxorubicin and cisplatin to induce cleavage of caspase-8, caspase-9 and poly(ADP-ribose) polymerase**. MDA-MB-231 and BT-20 cells were treated with doxorubicin (DOXO) or cisplatin (CDDP) alone or in combination with α-TEA for 24 hours. Western blot analyses were used to detect poly(ADP-ribose) polymerase (PARP), caspase-8, and caspase-9 cleavage **(a), (b)**. Data are representative of at least two independent experiments. GAPDH, glyceraldehyde-3-phosphate dehydrogenase; VEH, vehicle.

Based on the half-maximal effective concentration values for apoptosis presented in Table 2, the MDA-MB-231 and BT-20 cell lines that are more resistant to DOXO and CDDP were chosen to study the combinational effects of α-TEA + DOXO or α-TEA + CDDP on apoptosis induction. Data showed that α-TEA at 10 and 20 μM significantly enhanced apoptosis in combination with DOXO and CDDP in MDA-MB-231 and BT-20 cells, respectively, in comparison with individual treatments (Figure 1a to 1d). The mean combination index for the combination of α-TEA + DOXO was 0.41 ± 0.07 and 0.53 ± 0.05 for MDA-MB-231 and BT-20 cells, respectively (Table 3). The mean combination index for the combination of α-TEA + CDDP was 0.45 ± 0.10 and 0.75 ± 0.08 in MDA-MB-231 and BT-20 cells, respectively (Table 3). These data demonstrate that combinations of α-TEA + DOXO or α-TEA + CDDP synergistically induce apoptosis in both cell lines. Western blot analyses show that α-TEA at 20 μM cooperates with DOXO and CDDP to induce elevated levels of cleaved caspase-8, caspase-9, and PARP in both cell lines (Figure 2a,b), indicating that apoptosis induced by these combinations involves both caspase-8 and caspase-9 activation.

**Table 3:** Combination index of apoptosis

		Combination index^a^				
		
Cell line	α-TEA:drug^b^	ED_50_	ED_75_	ED_90_	Mean ± SD^c^	
DOXO						
MDA-MB-231	20:1	0.48	0.40	0.35	0.41 ± 0.07	Synergism^d^
BT-20	20:1	0.48	0.52	0.58	0.53 ± 0.05	Synergism
CDDP						
MDA-MB-231	2:1	0.55	0.44	0.36	0.45 ± 0.10	Synergism
BT-20	4:1	0.68	0.74	0.84	0.75 ± 0.08	Synergism

MDA-MB-231 and BT-20 breast cancer cells were treated with different concentrations of α-TEA, doxorubicin (DOXO), and cisplatin (CDDP) alone and in combination for 24 hours. Apoptosis was determined using annexin V-FITC/PI staining/fluorescence-activated cell sorter assay as described in Material and methods. ^a^For each combination treatment, a combination index was calculated using commercially available software (Calcusyn; Biosoft, Manchester, UK). ^b^The ratio for the concentrations used in combination treatments was determined from the data in Figure 1. ^c^The mean ± standard deviation (SD) calculated from the combination index values of the effective dose in 50% (ED_50_), the effective dose in 75% (ED_75_) and the effective dose in 90% (ED_90_) of the population. ^d^Combination index value: <1.0, synergism; 1.0, additive effect; >1.0, antagonism.

### p73 protein level is upregulated by α-TEA + DOXO or α-TEA + CDDP combinations and is involved in combination-induced apoptosis

**Figure 3:**
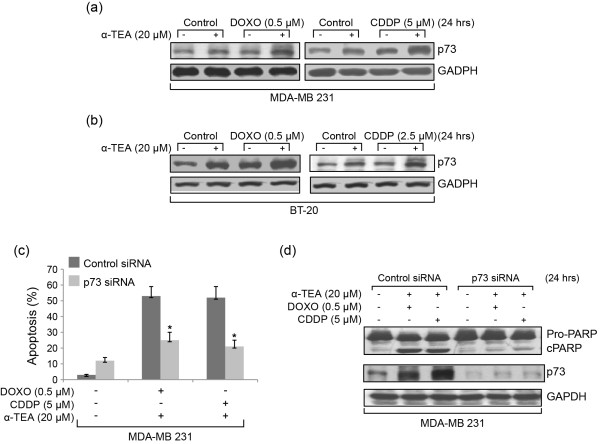
**p73 is upregulated by combination treatments and involved in combination-induced apoptosis**. MDA-MB-231 and BT-20 cells were treated with α-TEA, doxorubicin (DOXO) and cisplatin (CDDP) alone or in combination for 24 hours. Western blot analyses were performed to detect protein levels of p73 with glyceraldehyde-3-phosphate dehydrogenase (GAPDH) serving as loading control **(a), (b)**. MDA-MB-231 cells were transfected with p73 siRNA or control siRNA for 2 days and treated with combinations of α-TEA + DOXO or α-TEA + CDDP for 24 hours. Fluorescence-activated cell sorter/annexin V assays were used to determine the percentage of apoptotic cells **(c)**. Western blot analyses were used to verify the knockdown efficiency of p73 siRNA and the effect of p73 siRNA on combination-induced poly(ADP-ribose) polymerase (PARP) cleavage **(d)**. Data in (a), (b) and (d) representative of at least two independent experiments; data in (c) expressed as mean ± standard deviation from three independent experiments. *P < 0.05, significantly different from control siRNA determined by *t *test.

Since DOXO and CDDP as well as α-TEA have been shown to induce p73 upregulation in breast cancer cells [11,17,23], the combination of α-TEA + DOXO or α-TEA + CDDP was investigated for ability to cooperatively enhance p73 protein expression. Single treatments with DOXO, CDDP or α-TEA at sub-apoptotic levels for 24 hours slightly increased p73 protein expression above control levels, whereas combinations at the same levels markedly enhanced p73 protein expression in comparison with single treatments in both MDA-MB-231 and BT-20 cells (Figure 3a,b). siRNA to p73 significantly reduced the ability of combination treatments to induce apoptosis as determined by annexin V (Figure 3c) and PARP analyses (Figure 3d) in MDA-MB-231 cells. Western blot data show that siRNA to p73 effectively silenced p73 protein expression (Figure 3d). These data indicate that p73 activation by combination treatments is critical for induction of cell death by apoptosis.

### Combinations of α-TEA + DOXO or α-TEA + CDDP upregulate pro-apoptotic and downregulate anti-apoptotic mediators at both mRNA and protein levels

**Figure 4:**
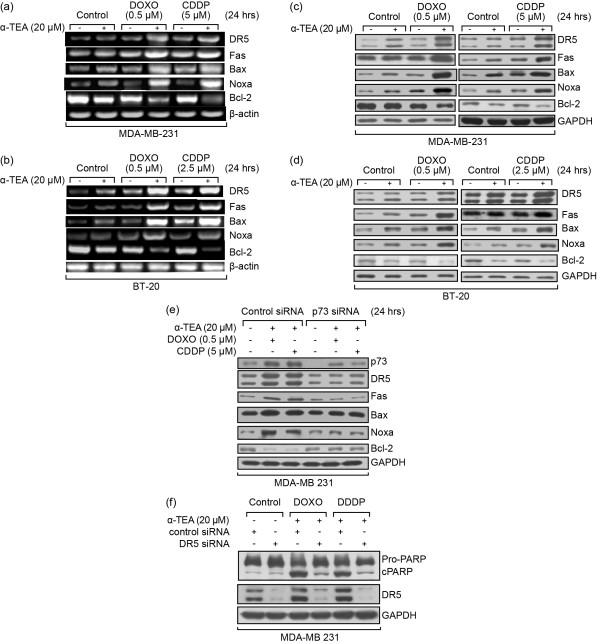
**α-TEA cooperates with doxorubicin or cisplatin to upregulate pro-apoptotic and downregulate anti-apoptotic mRNAs and proteins**. α-TEA cooperates with doxorubicin (DOXO) or cisplatin (CDDP) to upregulate mRNAs and proteins of the pro-apoptotic mediators death receptor 5 (DR5), Fas, Bax, and Noxa, and downregulate anti-apoptotic Bcl-2 mRNA and protein, all of which are downstream targets of p73. MDA-MB-231 and BT-20 cells were treated with DOXO or CDDP alone or in combination with α-TEA for 24 hours. mRNA levels of DR5, Fas, Bax, Noxa and Bcl-2 were determined by RT-PCR with β-actin serving as loading control **(a), (b)**. Protein levels of DR5, Fas, Bax, Noxa and Bcl-2 were determined by western blot analyses with glyceraldehyde-3-phosphate dehydrogenase (GAPDH) serving as loading control **(c), (d)**. The same treated samples as Figure 3d were used to detect the effect of siRNA to p73 on the combination-induced increase in protein levels of DR5, Fas, Bax, Noxa and decrease in Bcl-2 by western blot analyses with GAPDH as loading control **(e)**. MDA-MB-231 cells were transfected with DR5 siRNA or control siRNA for 2 days and treated with combinations of α-TEA + DOXO or α-TEA + CDDP for 24 hours. Western blot analyses were used to determine the effect of siRNA to DR5 on combination-induced poly(ADP-ribose) polymerase (PARP) cleavage and to verify the knockdown efficiency of DR5 **(f)**. Data representative of at least two independent experiments.

Published data show that p73 can regulate p53-dependent genes in p53-deficient cells [11]. To better understand the cellular events involved in p73-mediated apoptosis in combination treatments, mRNA and protein expression of p53-mediated pro-apoptotic mediators DR5, Fas, Bax, and Noxa, and anti-apoptotic mediator Bcl-2 were examined. Combinations of α-TEA + DOXO or α-TEA + CDDP enhanced DR5, Fas, Bax and Noxa mRNA (Figure 4a,b) and protein expression (Figure 4c,d), and decreased Bcl-2 mRNA (Figure 4a,b) and protein expression (Figure 4c,d) in MDA-MB-231 and BT-20 cells. siRNA knockdown of p73 was performed to determine whether expression levels of these mediators were regulated by p73. siRNA to p73 in MDA-MB-231 cells effectively silenced p73 protein expression and blocked the ability of combinations to induce increased levels of DR5, Fas, Bax and Noxa protein, as well as to decrease Bcl-2 protein levels (Figure 4e). These data suggest that combination treatments induce upregulation of pro-apoptotic mediators and downregulation of an anti-apoptotic mediator in a p73-dependent manner in p53 mutant, TNBC MDA-MB-231 and BT-20 cells. Recent studies in our laboratory show that DR5 pro-apoptotic signaling contributes to α-TEA-induced apoptosis [19,20]. To determine whether DR5 contributes to combination treatment-induced apoptosis, DR5 was functionally knocked-down with siRNA. Data indicate that silencing DR5 protein expression blocks combination-induced apoptosis as determined by PARP cleavage (Figure 4f).

### α-TEA cooperates with DOXO or CDDP to upregulate pc-Abl and pJNK, upstream mediators of p73

**Figure 5:**
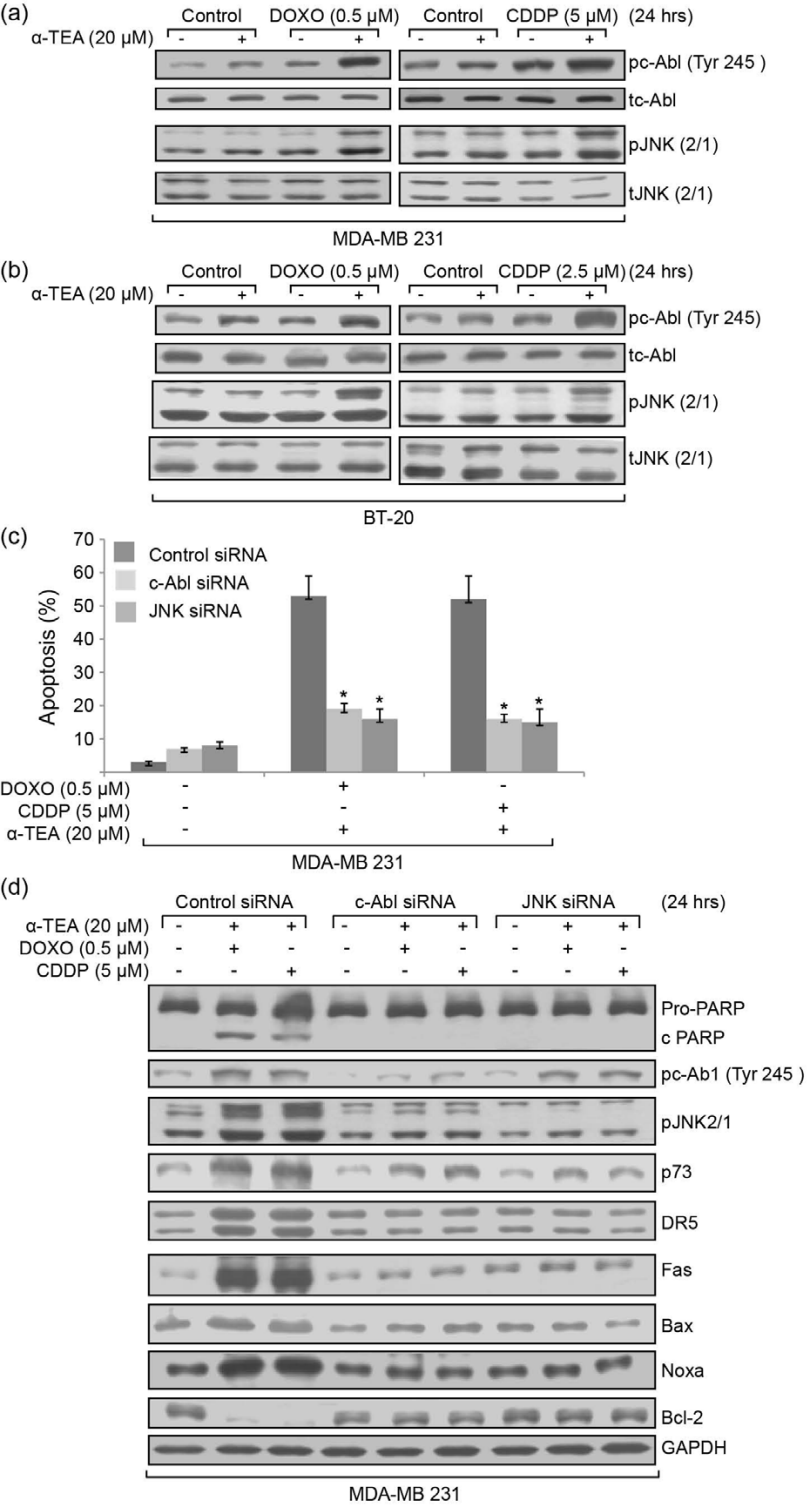
**α-TEA cooperates with doxorubicin or cisplatin to upregulate pc-Abl and pJNK, which can serve as upstream mediators of p73**. α-TEA cooperates with doxorubicin (DOXO) or cisplatin (CDDP) to upregulate pc-Abl and pJNK, which can serve as upstream mediators of p73. MDA-MB-231 and BT-20 cells were treated with DOXO or CIDDP alone or in combination with α-TEA for 24 hours. Protein levels of pc-Abl (Tyr-245), total c-Abl (tc-Abl), pJNK2/1, and total JNK2/1 (tJNK2/1) were determined by western blot **(a), (b)**. MDA-MB-231 cells were transfected with c-Abl and JNK siRNAs, as well as control siRNA for 2 days and treated with a combination of α-TEA + DOXO or α-TEA + CDDP for 24 hours. Apoptosis was determined by annexin V/fluorescence-activated cell sorter **(c)**. Western blot analyses were used to verify the knockdown efficiency of c-Abl and JNK siRNAs and the effect of c-Abl and JNK siRNAs on combination-induced poly(ADP-ribose) polymerase (PARP) cleavage, as well as p73 and p73-mediated death receptor 5 (DR5), Fas, Bax, Noxa and Bcl-2 **(d)**. Data in (a), (b), and (d) representative of at least two independent experiments; data in (c) expressed as mean ± standard deviation from three independent experiments. *P <0.05, significantly different from control siRNA determined by *t *test. GAPDH, glyceraldehyde-3-phosphate dehydrogenase.

Studies show that p73 can be upregulated upon DNA damage via activation of c-Abl and JNK [23,24]. To understand how p73 is activated by the combination treatments, phosphorylated levels of c-Abl and JNK2/1 were examined. Combinations of α-TEA + DOXO or α-TEA + CDDP induced increased levels of pc-Abl (Tyr-245) and pJNK2/1 in both cell lines (Figure 5a,b). siRNA knockdown of c-Abl or JNK significantly reduced the ability of combination treatments to induce apoptosis in MDA-MB-231 cells as determined by annexin V (Figure 5c) and PARP cleavage (Figure 5d). siRNA treatments blocked the ability of combination treatments to increase protein levels of p73 and blocked the ability of combination treatments to increase protein levels of DR5, Fas, Bax and Noxa, and to decrease the level of Bcl-2 (Figure 5d). siRNA to c-Abl blocked the ability of combination treatments to induce increased levels of pJNK, whereas siRNA to JNK had no effect on the ability of combination treatments to induce increased levels of pc-Abl (Tyr 245) (Figure 5d). These data show that activation of p73 is mediated by c-Abl and JNK in the combination treatments, and suggest that c-Abl, in part, regulates the phosphorylation status of JNK.

### Yap is involved in combination-induced apoptosis

**Figure 6:**
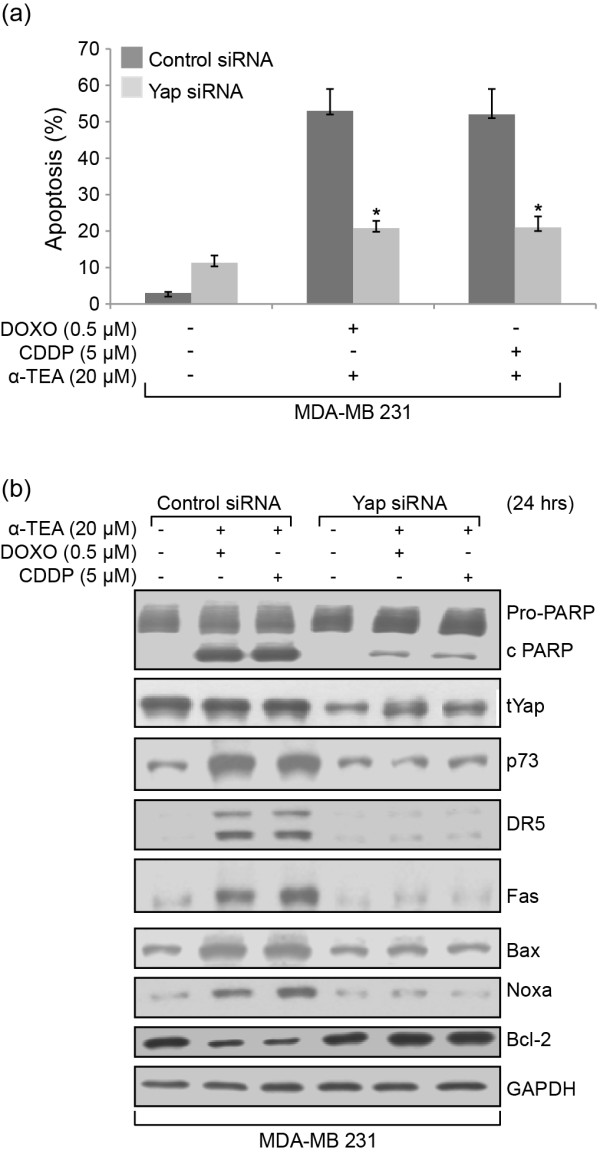
**Yap is involved in combination treatment-induced apoptosis**. MDA-MB-231 cells were transfected with Yap siRNA or control siRNA for 2 days and treated with combinations for 24 hours. Apoptosis was determined by annexin V/fluorescence-activated cell sorter **(a)**. Western blot analyses were used to verify the knockdown efficiency of Yap siRNA and the effect of Yap siRNA on combination-induced poly(ADP-ribose) polymerase (PARP) cleavage, as well as p73 and p73-mediated death receptor 5 (DR5), Fas, Bax, Noxa and Bcl-2 **(b)**. Data (a) expressed as mean ± standard deviation from three independent experiments; data in (b) representative of at least two independent experiments. *P <0.05, significantly different from control siRNA determined by *t *test. CDDP, cisplatin; DOXO, doxorubicin; GAPDH, glyceraldehyde-3-phosphate dehydrogenase.

Since Yap, a transcriptional co-activator Yes-associated protein, can interact with p73, resulting in enhanced p73 transcriptional activity [25] and stability [26,27], we determined whether Yap contributes to combination-induced apoptosis and increased p73 expression. siRNA knockdown of Yap significantly reduced the ability of combination treatments to induce apoptosis as measured by annexin V analyses (Figure 6a) and western blot analyses of PARP cleavage (Figure 6b). siRNA to Yap effectively reduced Yap protein levels and blocked combination treatment effects on p73 protein expression, as well as combination effects on DR5, Fas, Bax, Noxa and Bcl-2 protein expression (Figure 6b). These data show that Yap is a key player in combination treatment-induced apoptosis mediated by p73.

### Combination treatments induce Yap nuclear translocation, which is associated with suppression of phosphorylation of Akt and Yap

Yap activity can be regulated by c-Abl via phosphorylation of Yap at Tyr-357, leading to its stabilization and higher affinity for p73 [28,29]. Furthermore, Yap can be negatively regulated by Akt [29,30]. Akt induces Yap phosphorylation at Ser-127, resulting in Yap cytosolic localization via promoting Yap binding with 14-3-3, resulting in inactivation of Yap [30]. Since α-TEA has been shown to decrease pAkt in prostate cancer cells [15], ovarian cancer cells [18], and breast cancer cells (data not shown) we examined the effect of combination treatments on Yap nuclear translocation, as well as on pAkt and pYap expression.

**Figure 7:**
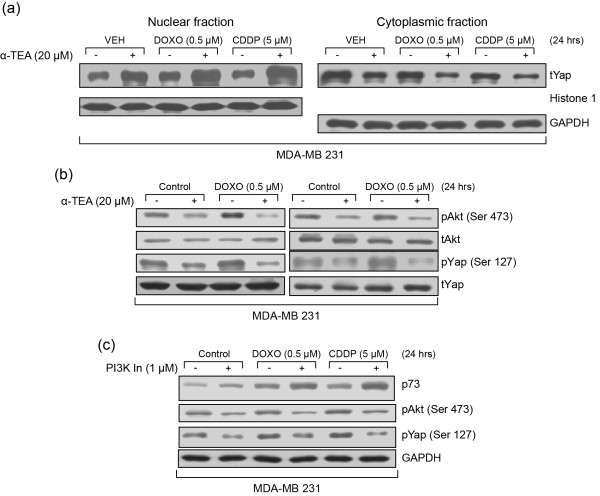
**Combination treatments induce Yap nuclear translocation, associated with suppression of phosphorylation of Akt and Yap**. Isolated cytosolic and nuclear fractions from MDA-MB-231 cells treated with α-TEA + doxorubicin (DOXO) or α-TEA + cisplatin (CDDP) were used to detect Yap tanslocation from cytosol to the nucleus by western blot analyses **(a)**. MDA-MB-231 cells were treated with DOXO or CDDP alone or in combination with α-TEA for 24 hours. Protein levels of pAkt and pYap were determined by western blot analyses with total Akt and Yap serving as controls **(b)**. MDA-MB-231 cells were pre-treated with Akt inhibitor wortmannin at 1 μM or dimethylsulfoxide for 2 hours followed by treatments with DOXO or CDDP for 24 hours. Protein levels of p73, pAkt and pYap were determined by western blot analyses **(c)**. Data are representative of at least two independent experiments. GAPDH, glyceraldehyde-3-phosphate dehydrogenase; VEH, vehicle.

Combination treatments of MDA-MB-231 cells induced increased levels of Yap protein in the nuclear fraction and reduced levels of Yap protein in the cytoplasmic fraction. Histone 1 and glyceraldehyde-3-phosphate dehydrogenase were used to evaluate the purity of nuclear and cytoplasmic fractions, respectively, and served as lane load controls (Figure 7a). Furthermore, data show that DOXO and CDDP increased pAkt and pYap protein expression, while α-TEA cooperated with DOXO or CDDP to suppress pAkt and pYap in MDA-MB-231 (Figure 7b). These data suggest that Yap nuclear translocation may partially contribute to p73-mediated effects and that combination treatment downregulation of pAkt correlates with decreased levels of pYap. To assess the role of Akt in DOXO-induced and CDDP-induced p73 protein expression, we examined the impact of phosphoinositide 3-kinase/Akt inhibitor (wortmannin) on DOXO-induced and CDDP-induced p73 protein expression. Data show that wortmannin enhanced DOXO-induced and CDDP-induced upregulation of p73 protein expression (Figure 7c), indicating a role for Akt in DOXO and CDDP increase in p73 expression. Data also show that wortmannin blocked DOXO-induced and CDDP-induced upregulation of pAkt and pYap (Figure 7c), suggesting that suppression of pAkt enhances DOXO-induced and CDDP-induced p73 expression via downregulation of pYap.

## Discussion

p73 is an important target for treating p53 mutant cancers [10,31-33]. The novel findings in the present study are as follows. First, α-TEA - a potent anticancer analog of vitamin E - synergizes with DNA-damaging agents DOXO and CDDP to induce apoptosis of human p53 mutant, triple-negative human breast cancer MDA-MB-231 and BT-20 cells via targeting p73. Second, combination treatments result in p73-dependent upregulation of pro-apoptotic DR5, Fas, Bax and Noxa, and downregulation of anti-apoptotic mediator Bcl-2 - all of which are p53-mediated apoptotic-related genes. Third, p73 and p73-mediated apoptotic events are regulated by c-Abl, JNK and Yap in combination treatments. Finally, α-TEA downregulation of Akt partially contributes to p73 upregulation in combination treatments. Our data therefore, for the first time, identify α-TEA as a small bioactive anticancer agent that regulates p53-mediated genes via p53-independent mechanisms when combined with DNA-damaging agents.

As a transcription factor, p73 shares structural and functional similarities with p53 [9,32,33]. In cancer cells that express wildtype p53, p73 has been reported to cooperate with p53 to induce apoptosis [34]; whereas in p53 mutant cancer cells, p73 has been reported to induce apoptosis via activation of p53-inducible genes [11,35]. Typically, p53 induces apoptosis via regulating apoptosis-related genes such as DR5, Fas, Bax, Noxa and Bcl-2 [36,37]. p73 is upregulated in response to a subset of DNA-damaging agents, including DOXO, CDDP, camptothecin and etoposide [38]. Several p53-mediated apoptosis-related genes have been identified to be regulated by p73, such as Fas, Bax, Bim, Noxa and Puma [17,39-41]. Whether DR5 is a direct target of p73, however, is not well documented. It has been reported that DR5 is regulated by p73 in H1299 human nonsmall lung cancer cells [42]. El-Deiry and coworkers used a high-throughput screen to identify small molecules that could activate p53 reporter activity, increase expression of p53 target genes such as p21(Waf1), DR5 and TRAIL, and induce apoptosis in p53-deficient colon cancer cells [35]. Some of these compounds activated a p53 response by increasing p73 expression, and knockdown of p73 with siRNA reduced their ability to activate p53 reporter activity while other compounds acted in a p73-independent fashion [35]. In addition, they characterized a derivative of the plant alkaloid ellipticine as an anticancer agent that induces p73 and DR5 protein expression in a p53-deficient human colon carcinoma cell line [43]. Neither of these studies, however, showed direct evidence that p73 was regulating DR5 transcription. To the best of our knowledge, there is no direct evidence showing that p73 regulates DR5 transcription other than the lung cancer studies [42]. In addition, there is no evidence to indicate that p73 transcriptionally regulates Bcl-2. The present study thus demonstrates, for the first time, that both DR5 and Bcl-2 are mediated at the transcriptional level by p73 in p53 mutant, TNBC MDA-MB-231 and BT-20 human breast cancer cells treated with α-TEA combined with DOXO or CDDP as determined by siRNA knockdown assays. Our previous data showed that DR5 is involved in α-TEA-induced apoptosis since siRNA knockdown of DR5 blocked α-TEA-induced apoptosis in MCF-7 and MDA-MB-231 human breast cancer cells [19,20]. Here, we demonstrated that DR5 is necessary, at least in part, for apoptosis induced by α-TEA combination treatments with DOXO or CDDP.

Besides transcriptionally activating p53-mediated apoptotic genes, p73 has been reported to induce ER stress via transactivation of Scotin [44]. Since DR5 and Bcl-2 expression can be regulated by ER stress via CHOP [45,46], and since α-TEA has been shown to induce ER stress and CHOP expression [19], we cannot rule out the possibility that p73 regulates DR5 and Bcl-2 via ER stress in combination treatments. Further studies are needed to address this issue.

p73 is predominantly regulated at the post-translational level in response to DNA-damaging agents. c-Abl and JNK are activated by DNA-damaging agents and both are involved in p73 activation [23,24]. DOXO and CDDP have been shown to regulate p73 via c-Ab1 [23,28,29]. c-Abl regulates p73 via different mechanisms; for example, c-Abl can directly stabilize p73 via acetylation and phosphorylation of p73 [23,47], and can stabilize p73 and enhance p73 transcriptional activity via phosphorylation of Yap [28]. JNK has been reported to stabilize p73 via phosphorylation of p73 [24] and via JNK phosphorylation/activation of c-Jun [48]. In addition, JNK also activates p73 via enhancing c-Abl nuclear translocation [49]. In untreated cells, c-Abl is sequestered in the cytosol by 14-3-3 proteins. Upon exposure of cells to DNA damaging agents, JNK is activated and phosphorylates 14-3-3, resulting in the release of c-Abl into the nucleus, an event required for the induction of apoptosis in response to DNA-damaging agents [49]. Published data [50] and the present data show that c-Abl also regulates JNK via phosphorylation, suggesting cross-talk between c-Abl and JNK.

Yap is a transcriptional coactivator, which can interact with the p53 family member p73, resulting in an enhancement of p73's transcriptional activity [25,26] and stability [27]. A potential mechanism of the p73 protein stabilization was recently suggested by Levy and colleagues [27]. Namely, Yap competes with Itch, an E3 ubiquitin ligase involved in degradation of p73, for binding to p73 at the PPXY motif. Furthermore, Yap activity can be regulated by c-Abl via phosphorylation at Tyr-357, leading to a more stable form of Yap that exhibits a higher affinity to p73 [27]. Yap can be negatively regulated by Akt [30,51]. Akt induces Yap phosphorylation at Ser-127, resulting in Yap cytosolic localization since phosphorylation of Yap at Ser-127 promotes Yap binding with 14-3-3 [30]. Yap activation can thus be regulated in a positive manner by c-Abl and in a negative manner by Akt. DNA damage can activate survival mediator Akt, resulting in reducing the anticancer efficacy of DNA-damaging drugs. DOXO or CDDP induces activation of Akt in some cell lines [52,53]. Likewise, our data show that DOXO and CDDP induce elevated levels of pAkt not only in MDA-MB-231 cells (Figure 7), but also in MCF-7, MDA-MB-453 and BT-20 cells (data not shown). As expected, Akt inhibitors have been reported to enhance the anticancer effect of DOXO in MDA-MB-231 cells [54]. Data reported here show that Akt inhibitor wortmannin enhanced DOXO-mediated and CDDP-mediated increases in p73 protein expression, which is associated with downregulation of pAkt and pYap (Ser-127) in MDA-MB-231 cells. Taken together, these data suggest that Akt activation upon DNA damage may counteract p73 activation induced by JNK and c-Abl via inhibition of Yap nuclear translocation. Our data thus suggest that Yap nuclear translocation plays an important role in p73 activation and that suppression of pAkt and its inhibitory phosphorylation of pYap contributes to enhanced Yap nuclear translocation in combination treatments.

How α-TEA induces p73 protein expression is not fully understood. We previously reported that JNK is involved in regulation of p73 in α-TEA-induced apoptosis of human breast cancer cells [19]. In the present study, we found that α-TEA also induces increased levels of pc-Abl and Yap nuclear translocation, as well as suppresses pAkt and pYap, suggesting that c-Abl and Yap, as well as downregulation of pAkt/pYap, are also involved in α-TEA-induced apoptosis. Noxa has been identified as a downstream mediator of p73 in α-TEA-induced apoptosis [17]. Whether other p53-mediated genes, such as Fas, DR5, Bax and Bcl-2, are regulated by 73 following α-TEA treatment, however, has not been investigated. Since recent data show that ER stress-mediated CHOP contributes to α-TEA-induced upregulation of DR5 and downregulation of Bcl-2 [19], it will be important for future studies to address whether both CHOP and p73 contribute to DR5 upregulation and Bcl-2 downregulation in α-TEA-induced apoptosis.

**Figure 8:**
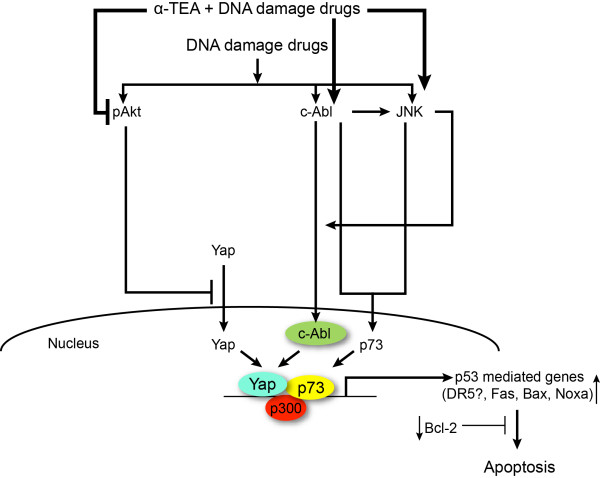
**Proposed signaling pathways**. Proposed pathways whereby the combination of α-TEA + doxorubicin (DOXO) or α-TEA + cisplatin (CDDP) induces apoptosis in p53 mutant, triple-negative MDA-MB-231 and BT-20 human breast cancer cells. p73 can be activated via multiple mechanisms and pathways, including: c-Abl and JNK can directly phosphorylate p73 to stabilize it; Yap in nucleus can bind with p73 to enhance its transcriptional activity and stability; and p73 can be transcriptionally regulated (not studied here). Yap can be regulated in a positive manner by c-Abl via phosphorylation enhancing its stability and transcriptional activity, and in a negative manner by Akt via inhibiting translocation of Yap into the nucleus. c-Abl can directly phosphorylate JNK and JNK can enhance c-Abl nuclear translocation. Therefore, c-Abl, JNK and Yap play positive roles and Akt plays a negative role in p73 activation. Our data show that DNA-damaging drugs DOXO and CDDP activated c-Abl and JNK, but also activated Akt, which can counteract c-Abl and JNK effects on activation of p73. Combination treatments not only act cooperatively to activate c-Abl and JNK, but also act cooperatively to inhibit pAkt and pYap (ser-127), leading to Yap nuclear translocation and p73 activation. In summary, combinations of α-TEA + DOXO or α-TEA + CDDP act cooperatively to upregulate c-Abl/JNK, induce Yap nuclear translocation and downregulate pAkt/pYap, leading to activation of p73 and upregulation of p73-mediated pro-apoptotic factors mediators, and downregulation of Bcl-2, thereby restoring DOXO and CDDP chemotherapeutic potential in p53 mutant, triple-negative breast cancers. DR5, death receptor 5.

Mechanisms mediating the combined anticancer effects of α-TEA + DOXO or α-TEA + CDDP are diverse and not completely understood. These studies identified p73 as a key player in combination treatment-induced apoptosis. In addition, data show that c-Abl, JNK and Yap play roles in combination treatment-induced activation of p73. It is important to note that although both α-TEA and DNA-damaging drugs DOXO or CDDP induce increased levels of pc-Ab1 and pJNK, only α-TEA and the combination of α-TEA + DOXO or α-TEA + CDDP induce Yap nuclear translocation, which is associated with inhibition of pAkt (Ser-473) and Akt-phosphorylated pYap (Ser-127). Furthermore, a phosphoinositide 3-kinase/Akt inhibitor was shown to enhance DOXO and CDDP upregulation of p73, which was also associated with downregulation of pAkt and pYap. Taken together, these data suggest that downregulation of pAkt and the pAkt-mediated inactive form of Yap play important roles in p73 activation and apoptosis in combination treatments. α-TEA thus cooperates with DOXO or CDDP to induce p73 protein expression and apoptosis not only via activation of c-Abl and JNK, but also via activation of Yap, which may be regulated positively by c-Abl and negatively by Akt. Based on published reports and the data presented here we proposed signaling events necessary for combination treatment-induced apoptosis in p53 mutant, TNBC cells (Figure 8).

## Conclusions

In summary, the data demonstrate that α-TEA, a small bioactive lipid, cooperates with DNA-damaging agents DOXO and CDDP to induce apoptosis in human breast cancer cells via targeting p53-inducible apoptotic-related genes in a p73-dependent manner. These studies highlight the potential for activation of p73 as a promising target for treatment of p53 mutant, TNBC and identify α-TEA as an important candidate agent.

## Abbreviations

α-TEA: RRR-α-tocopherol ether-linked acetic acid analog; CDDP: cisplatin; DOXO: doxorubicin; DR5: death receptor 5; ER: estrogen receptor; FBS: fetal bovine serum; FITC: fluorescein isothiocyanate; JNK: c-Jun N-terminal kinase; MEM: modified Eagle's medium; p-Akt: phospho-Akt; PARP: poly(ADP-ribose) polymerase; PCR: polymerase chain reaction; PI: propidium iodide; pJNK: phospho-c-Jun N-terminal kinase; RT: reverse transcriptase; siRNA: small interfering RNA; TNBC: triple-negative breast cancers; Yap: Yes-associated protein.

## Competing interests

US and international patents on α-TEA are held by the Research Development Foundation. KK, BGS and WY are listed as inventors. No commercial applications or financial gain have been realized.

## Authors' contributions

RT, WY, BGS and KK conceived and designed the study, analyzed the data, and drafted the manuscript. WY helped in cell culture study and RT performed all experiments.
